# Quantitative Phase Imaging with a Meta-Based Interferometric System

**DOI:** 10.1021/acsami.5c02901

**Published:** 2025-04-15

**Authors:** Cheng Hung Chu, Chen-Ming Tsai, Takeshi Yamaguchi, Yu-Xiang Wang, Takuo Tanaka, Huei-Wen Chen, Yuan Luo, Din Ping Tsai

**Affiliations:** △ YongLin Institute of Health, National Taiwan University, Taipei 10672, Taiwan; ‡ Institute of Medical Device and Imaging, National Taiwan University, Taipei 10051, Taiwan; § Innovative Photon Manipulation Research Team, RIKEN Center for Advanced Photonics, Saitama 351-0198, Japan; ∥ Program for Precision Health and Intelligent Medicine, National Taiwan University, Taipei 106319, Taiwan; ⊥ Metamaterials Laboratory, RIKEN Cluster for Pioneering Research, Saitama 351-0198, Japan; # Graduate Institute of Toxicology, College of Medicine, National Taiwan University, Taipei 100, Taiwan; ∇ Genome and Systems Biology Degree Program, National Taiwan University and Academia Sinica, Taipei 100, Taiwan; ○ Institute of Biomedical Engineering, National Taiwan University, Taipei 10051, Taiwan; ◆ Department of Electrical Engineering, City University of Hong Kong, Kowloon, Hong Kong 999077, China; ¶ Centre for Biosystems, Neuroscience, and Nanotechnology, City University of Hong Kong, Kowloon, Hong Kong 999077, China; ■ The State Key Laboratory of Terahertz and Millimeter Waves, City University of Hong Kong, Kowloon, Hong Kong 999077, China

**Keywords:** dielectric metasurface, digital holography, quantitative phase imaging, diffractive optics, interferometric microscopy

## Abstract

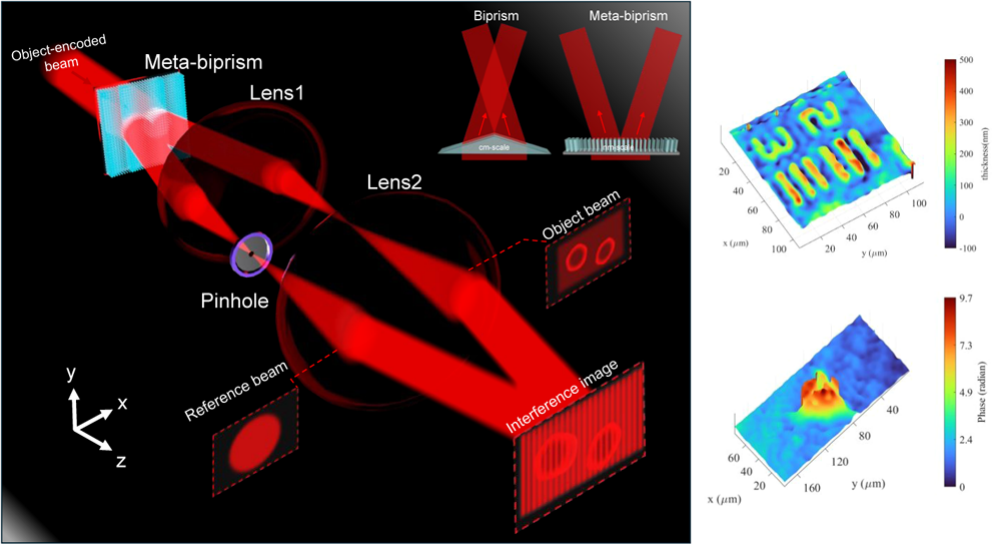

Optical phase imaging has become a pivotal tool in biomedical research, enabling label-free visualization of transparent specimens. Traditional optical phase imaging techniques, such as Zernike phase contrast and differential interference contrast microscopy, fall short of providing quantitative phase information. Digital holographic microscopy (DHM) addresses this limitation by offering precise phase measurements; however, off-axis configurations, particularly Mach–Zehnder and Michelson-based setups, are often hindered by environmental susceptibility and bulky optical components due to their separate reference and object beam paths. In this work, we have developed a meta-based interferometric quantitative phase imaging system using a common-path off-axis DHM configuration. A meta-biprism, featuring two opposite gradient phases created using GaN nanopillars selected for their low loss and durability, serves as a compact and efficient beam splitter. Our system effectively captures the complex wavefronts of samples, enabling the retrieval of quantitative phase information, which we demonstrate using standard resolution phase targets and human lung cell lines. Additionally, our system exhibits enhanced temporal phase stability compared to conventional off-axis DHM configurations, reducing phase fluctuations over extended measurement periods. These results not only underline the potential of metasurfaces in advancing the capabilities of quantitative phase imaging but also promise significant advancements in biomedical imaging and diagnostics.

## Introduction

Traditional bright-field microscopy plays a crucial role in various biomedical applications. It typically relies on the opacity of specimens to generate sufficient contrast for visual observation. In cases involving nearly transparent materials, such as biological cells and tissues, contrast enhancement is often achieved through fluorescence and staining methods. However, these approaches have inherent limitations, as fluorescent dyes and staining agents can negatively affect the viability of live cells.^1−3^ To address these challenges, label-free imaging modalities, such as Zernike phase contrast (ZPC) microscopy and differential interference contrast (DIC) microscopy, have been developed.^4−9^ These techniques convert phase variations within transparent samples into detectable intensity contrasts, offering benefits such as reduced phototoxicity and minimal sample preparation. Nonetheless, while these methods are effective in providing qualitative visualizations, they are inadequate for quantitative phase profile mapping, which is essential for comprehensive morphological and structural analysis.

Quantitative phase imaging (QPI) techniques, including quantitative differential phase contrast (qDPC) microscopy, Fourier ptychographic microscopy (FPM), transport-of-intensity equation (TIE), cross-grating wavefront microscopy (CGM) and digital holographic microscopy (DHM), have significantly enhanced the capabilities of label-free microscopy.^10−19^ These techniques enable the precise quantification of phase information in transparent specimens, thereby enriching our understanding of cellular structures. Notably, DHM excels in obtaining highly accurate quantitative phase information by capturing holographic interference patterns, which are subsequently processed through digital reconstruction. The standard DHM system uses either a Mach–Zehnder or Michelson interferometer, configured for transmission or reflection modes, and supports both on-axis (in-line) and off-axis setups.^20^ Although the on-axis configuration, which involves copropagating reference and object beams, is relatively straightforward, it suffers from image clarity issues due to the overlap of the zero-order (direct current, DC) term and the virtual image (twin images).^21^ In contrast, the off-axis configuration angles the reference and object beams with respect to each other, preventing overlap in the spatial frequency domain and thereby avoiding interference from the DC term and virtual image.^22^ This configuration enhances real-time phase and amplitude imaging, which is critical for dynamic observations of biological samples. However, the separation of the beam paths in the off-axis makes it highly susceptible to environmental fluctuations, resulting in significant phase noise and compromised spatial and temporal stability. Recent advancements have seen a rise in common-path off-axis configurations due to their lower cost, robustness, and stability, as both the signal and reference beams traverse through the same optical components and follow nearly identical optical paths.^23^ A key feature of these configurations is the use of a core optical element for beam splitting. Various optical elements have been proposed to fulfill this function, including Fresnel biprisms, diffraction gratings, and volume holographic grating, among others.^24−31^ Among these approaches, diffraction phase microscopy (DPM) is a well-established and effective common-path DHM configuration that utilizes a diffraction grating to generate the necessary reference and object beams from its zero-order and first-order diffraction components.^24^ However, conventional gratings introduce multiple diffraction orders, leading to power loss and unwanted signal contamination. As the demand for miniaturized optical systems continues to grow, ongoing research efforts in DHM are focused on developing more compact and flexible imaging solutions to address the limitations imposed by bulky traditional optical components.

Metasurfaces, ultrathin planar structures engineered from subwavelength features, are increasingly recognized for their unprecedented capability to precisely manipulate various degrees of freedom of electromagnetic waves, including phase, amplitude, polarization, and propagation direction.^32−45^ Notably, dielectric metasurfaces can be manufactured using semiconductor fabrication processes, which significantly lower production costs and enable large-scale production.^46−48^ By replacing bulky optical elements with compact, efficient designs, metasurfaces have revolutionized numerous optical applications, ranging from basic optics to advanced imaging and spectrometry, facilitating system miniaturization. Their potential in biomedical applications such as wide-field, tomographic, and endoscopic imaging, has garnered considerable attention, pushing the boundaries of traditional imaging modalities.^49−52^

Recent advances have seen the integration of metasurfaces into various phase contrast imaging techniques, marking a significant advancement. For example, an entirely metasurface-based spiral phase contrast imaging system has demonstrated, eliminating conventional optical elements while achieving compact and high-performance imaging.^53^ Another recent study has shown that spiral metalens, combining both focusing and phase contrast imaging functionalities into a single metasurface, can achieve submicrometer resolution across a broadband visible spectrum.^54^ The dynamic capabilities of metasurface are also demonstrated, such as the ability to switch between bright-field and edge-enhanced imaging, depending on the polarization state of the incident light.^55,56^ Furthermore, their feasibility in QPI techniques has been proven with implementations in technologies based on TIE, phase shifting, DHM, and so on.^57−65^ Despite the myriad advantages presented by metasurfaces, their integration into DHM remains relatively unexplored. Additionally, unlike TIE, phase-shifting methods, and qDPC, which typically require multiple images for phase reconstruction, DHM captures a full-field hologram in a single image. Moreover, its interferometric nature can offer the ability to digitally refocus and reconstruct different focal planes. Here we integrate the meta-biprism into the common-path off-axis DHM system to obtain the phase information on various objects. The ability to acquire and reconstruct complex optical fields with high stability is performed. Our results shed light on the advantages and prospective applications of metasurface in the ever-evolving world of imaging science.

## Results and Discussion

**Figure 1:**
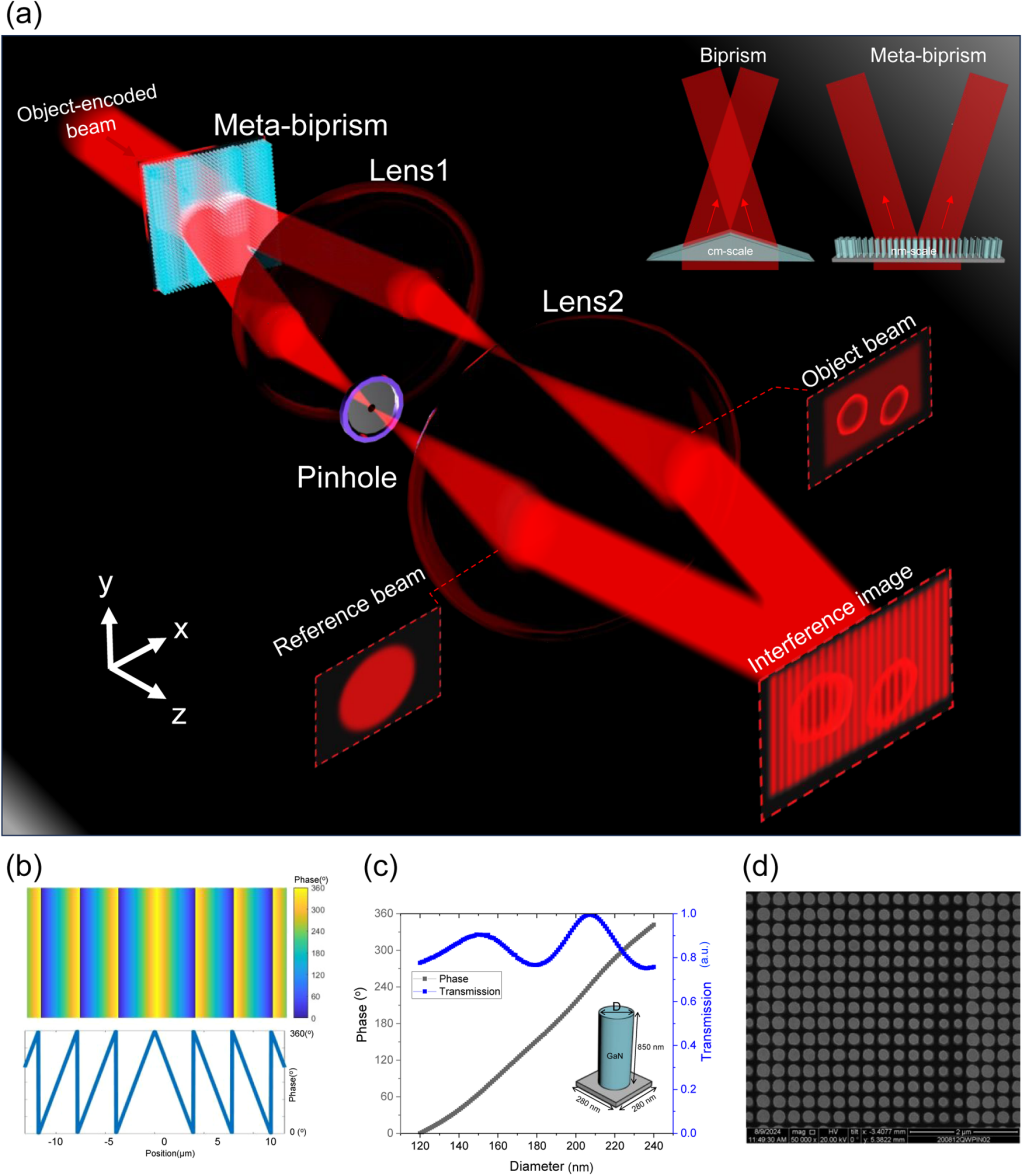
(a) Schematic of off-axis DHM based on meta-biprism. (b) Phase distribution in the center of meta-biprism. (c) Transmission and phase shift of GaN nanopillars calculated with a 1 nm step in diameter, with the lines formed from sampled data points corresponding to these parameters. (d) Top-view scanning electron microscopy (SEM) image of nanopillars.

A schematic of a metasurface-based common-path off-axis DHM system is presented in Figure 1a, illustrating how the meta-biprism separates an incident beam into two beams with distinct angles, ultimately leading to the formation of their interference pattern. One of these beams is filtered using a pinhole located at the Fourier plane of 4f-imaging system, which serves as the reference beam, while the other beam functions as the object beam, carrying information about the scattered complex field of the object. The beam-splitting capability of the meta-biprism arises from the introduction of two opposing gradient phase distributions, as shown in Figure 1b. The phase distribution is defined by the equation:
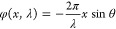
1

where *x* denotes the spatial coordinate, λ signifies the working wavelength, and θ represents the refraction angle. The angles θ are set at ± 9.28°, resulting in a phase distribution that corresponds to a conventional biprism with a 150° apex angle, as illustrated in Figure 1b. The optical phase modulation up to 2π at the working wavelength of 633 nm is achieved by arranging GaN nanopillars, each 850 nm in height and with diameters ranging from 120 to 240 nm, on a sapphire substrate, as shown in Figure 1c. Compared to a traditional biprism, this design not only reduces the thickness to the nanometer scale but also minimizes the optical path length by eliminating the need for the two beams to intersect, as depicted in the upper right inset. The nanopillars are fabricated using CMOS-compatible processes, including electron beam lithography followed by etching with inductively coupled plasma reactive ion (ICP-RIE). Further fabrication details are provided in the Methods section, and the SEM image of the fabricated nanopillars is shown in Figure 1d. The experimentally measured intensity distribution of the beam after passing through the meta-biprism along the *x*–*z* plane is presented in Figure S1. The two beams interfere off-axially on the image plane, positioned at the rear focal plane of a tube lens, and recorded by a CCD sensor (THORLABS, DCU223C). The complete optical setup is detailed in Figure S2.

The object beam (*O*) with an angle of θ and the reference beam (*R*) with an angle of −θ can be described as
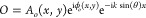
2
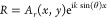
3The resulting interference pattern can be expressed mathematically as follows:
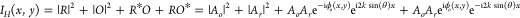
4where |*R*|^2^ represents the intensity of the reference beam, and |*O*|^2^ represents the intensity of the object beam. The interference terms R*O and RO*, representing the real and virtual images, respectively, involve the complex conjugates of the reference (R*) and object (O*) beams. The corresponding spectrum can be described as
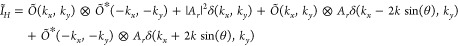
5where Õ denotes the Fourier transform of *O* and (*k*_*x*_, *k*_*y*_) are the coordinates in the frequency domain. The symbol ⊗ represents the convolution operation, and δ is the delta function. The first two terms of the equation correspond to the DC component of the hologram. The third term contains the actual object information, while the fourth term contains the conjugate of this information. The term δ*(k*_*x*_–*2k* sin(θ),*k*_*y*_*)* results in a shift of object signal in the frequency domain, enabling its separation from the conjugate term and the DC component. By isolating the third term and performing an inverse Fourier transform, we can accurately retrieve the complex field of the object beam.

**Figure 2:**
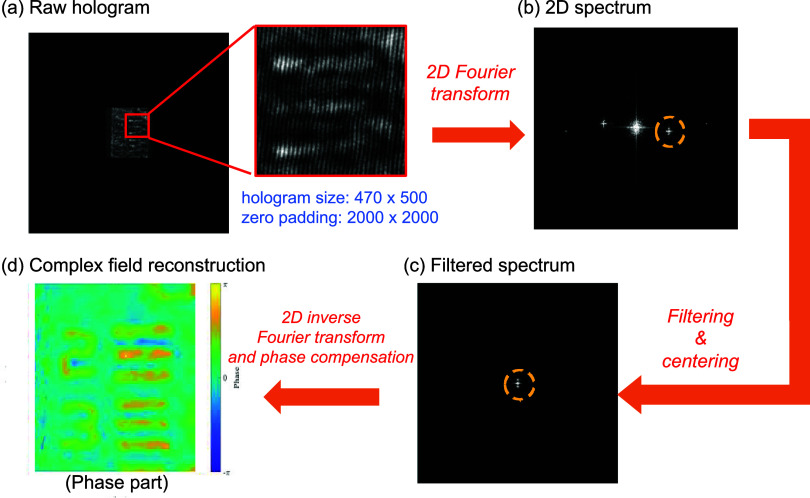
Reconstruction process of digital holography. (a) Recorded hologram of a transparent 1951 USAF resolution phase target with zero-padding to 2000 × 2000. (b) Corresponding 2D spectrum of the hologram. (c) Filtered and centered spectrum of the circled part in (b). (d) Reconstructed phase image.

The reconstruction of digital holography is achieved using the spatial filtering method, as shown in Figure 2. The raw hologram image of the 1951 USAF resolution phase target, which includes zero padding, is depicted in Figure 2a. Figure 2b illustrates its spatial spectrum by using 2D Fourier transformation. The spectrum reveals three distinct components: the zero, first, and minus first orders, corresponding to the DC, primary, and conjugate terms of the hologram, respectively. By applying a windowing operation within the Fourier domain, either the first or the minus first orders can be singled out, as shown in Figure 2c. This isolation enables the reconstruction of the object’s amplitude and phase information, effectively mitigating the impact of the DC term and the twin image effect. The offset angle between the signal and reference beams is crucial; a larger angle not only increases the fringe frequency but also shifts the spatial frequencies of image away from the DC term in the frequency domain. This shift enables clearer differentiation and more precise filtering in holographic reconstruction. However, capturing these high-frequency details without aliasing requires higher sampling rates. Achieving these higher spatial sampling rates of the sensor is often constrained by several factors, especially the resolution of the CCD (charge-coupled device) camera, as each pixel represents a sampling point. In our setup, the produced image has dimensions of 470 × 500 pixels, and CCD has a pixel pitch of 4.65 μm. According to the Nyquist theorem, the maximum allowable frequency is about 0.1075 cycles/μm. To illustrate the impact of different offset angles on holographic reconstruction, we conducted simulations, as shown in Figures S3–S5. Subsequently, the inverse Fourier transform yields the reconstructed phase information on the target. Background noise is discernible within the image, attributed to the tilt or unevenness of the sample, as well as disparities in the distribution of the light source. To address this, we implement a phase compensation methodology.^66^ By subtracting background noise, we achieve a quantitative phase image free from the interference of background artifacts, as shown in Figure 2d, with the detailed process illustrated in Figure S6.

**Figure 3:**
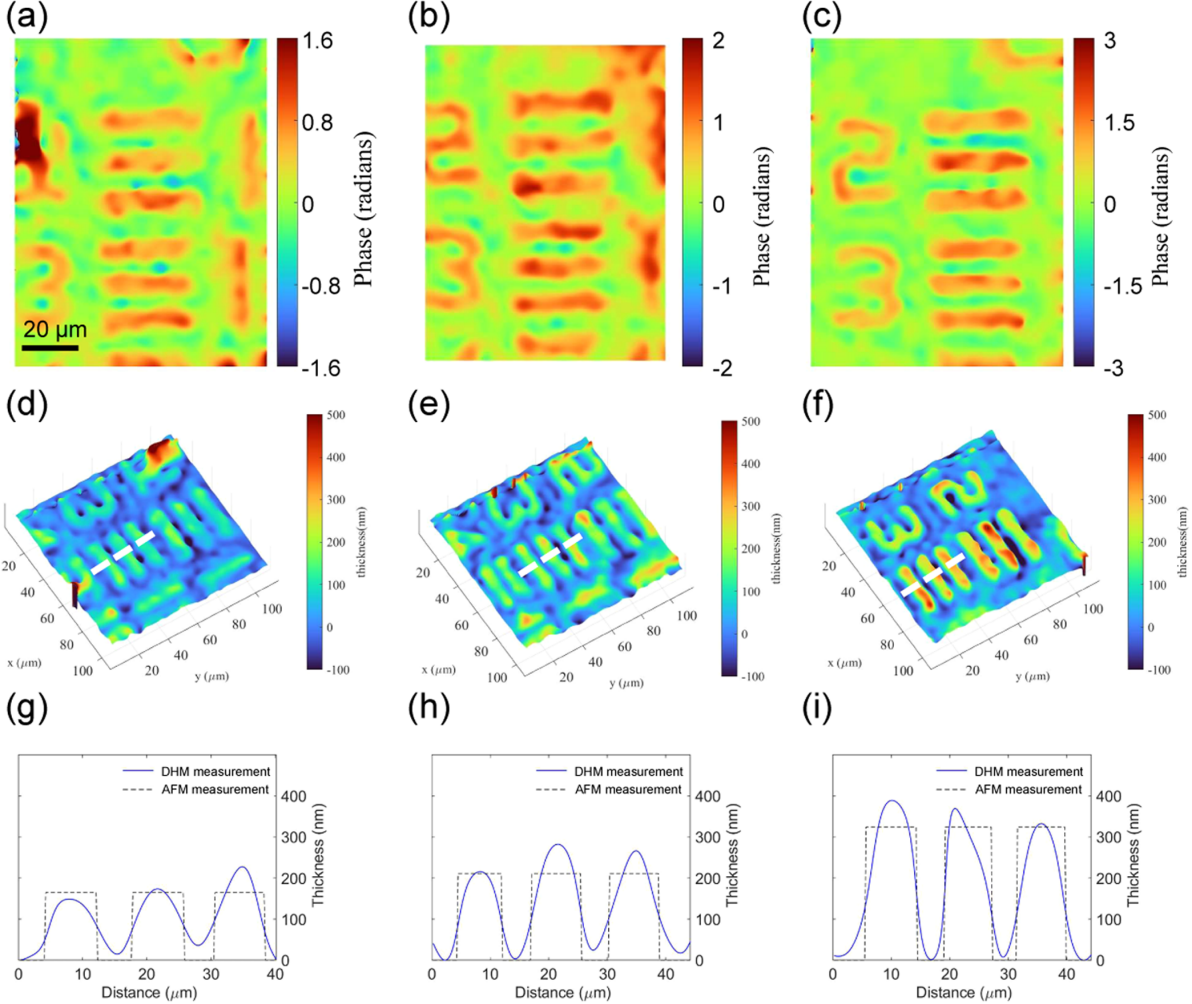
Retrieved phase of the third element in the fifth group (40.3 lp/mm) of phase target with different thicknesses of (a) 150 nm, (b) 200 nm, and (c) 300 nm, and (d–i) their corresponding 3D view. (g–i) Thickness distribution along the white dotted line in (d–f), with the blue line representing the measured thickness and the black dotted line denoting the theoretical thickness distribution.

The reconstructed phase images of the resolution phase target at 150, 200, and 300 nm in thickness are depicted in Figure 3. These images reveal a high-contrast depiction of the characteristics of the target, offering valuable quantitative phase information. The phase values obtained from these images represent the spatial distribution of phase discrepancies within the target, influenced by factors such as its thickness and the variation in refractive index relative to the surrounding medium. The thickness of samples can be calculated using the following equation:
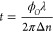
6

where ϕ*_o_* is the phase distribution of the sample, λ represents the working wavelength, and Δ*n* is the refractive index difference between the target and the ambient media, which is 1.52. The average thicknesses of the target structures measured by our DHM are 167, 243, and 369 nm, with corresponding Atomic Force Microscopy (AFM) measurements of 165.3, 211.4, and 324.7 nm, resulting in percentage differences of 0.45, 15.6, and 10.7%, respectively. Additionally, detailed three-dimensional information can be obtained from the complex amplitude of a captured hologram, enabling precise depth reconstruction. The capability is demonstrated in the Supporting Information (Figure S7), which includes the defocused hologram of the amplitude resolution target, its corresponding bright-field image, and the reconstructed complex field computed using the angular spectrum method.

**Figure 4:**
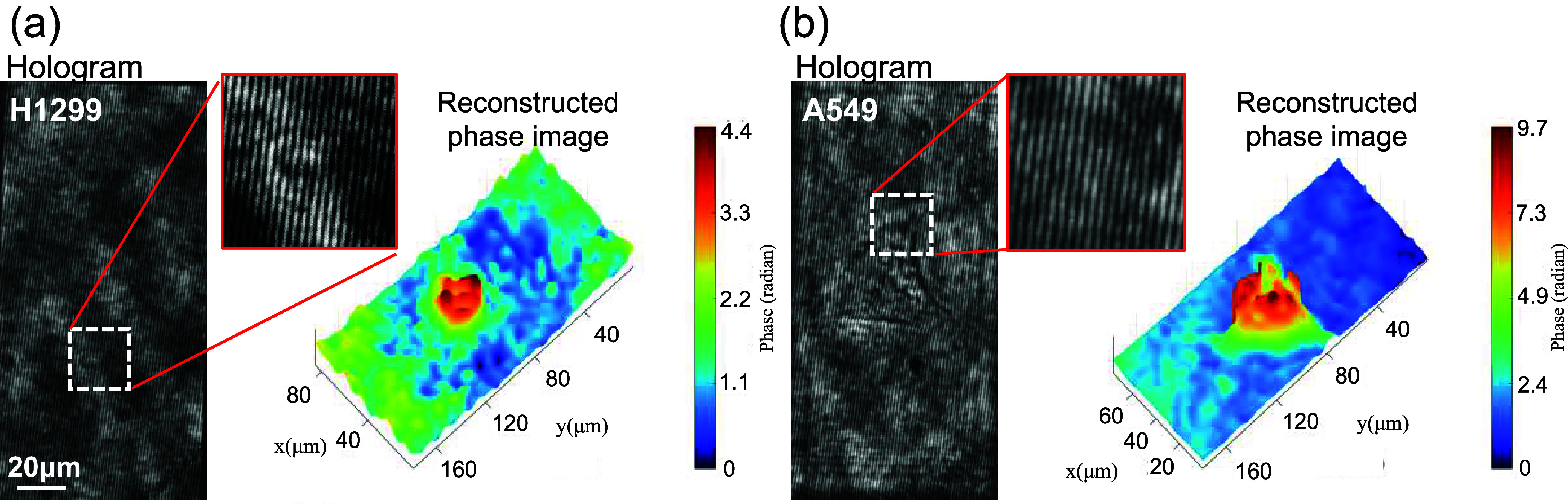
Reconstructed phase images of H1299 cells (a) and A549 cells (b), each obtained from separately prepared sample slides. Each slide contained many cells.

We experimentally validate the imaging capabilities of the proposed system using two human lung cell lines, H1299 and A549, as illustrated in Figure 4a,b. These figures show the raw hologram images of these cells alongside with their corresponding quantitative unwrapped phase images. H1299 cells represent a human lung adenocarcinoma cell line commonly utilized in cancer research, characterized by the absence of functional p53. In contrast, A549 cells are another lung adenocarcinoma line notable for retaining wild-type p53, making them complementary models for studying p53-related cancer mechanisms. Both cell lines are valuable for investigating lung cancer biology, drug screening, and resistance mechanisms. The cells are fixed on a glass slide, immersed in phosphate-buffered saline (PBS) with a refractive index of approximately 1.335, and mounted on the stage for hologram recording. Following phase reconstruction, we use a phase unwrapping algorithm to address the issue of cell thickness exceeding the 2π phase limit.^67^

**Figure 5:**
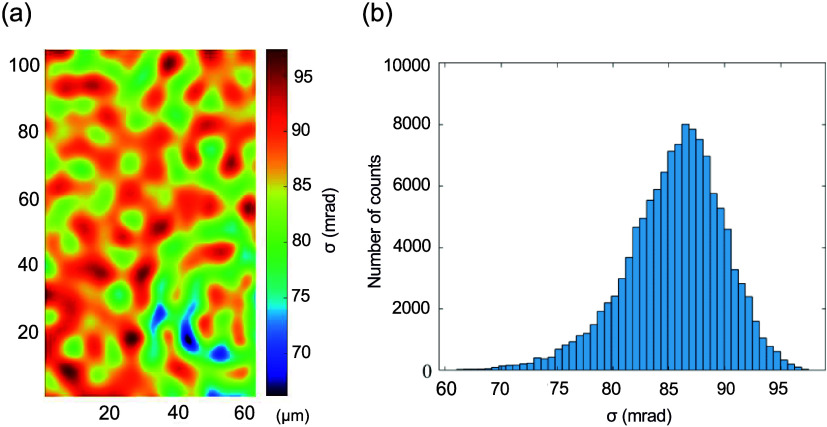
Temporal phase stability evaluation of our system. (a) Pixel-wise standard deviation map of temporal phase fluctuations, computed from 122,850 individual data points across 150 frames captured at 10 frames per second over 15 s. (b) Histogram illustrating the distribution of standard deviations shown in (a).

We also evaluated the temporal stability of our system using a blank glass slide as the test sample, with the results presented in Figure 5. A total of 150 holograms are captured at 10 frames per second over a 15 s duration. Each hologram consists of 273 × 450 pixels (122,850 individual data points), which are analyzed to compute the standard deviation (σ) of phase fluctuations. Figure 5a presents the pixel-wise standard deviation map across all frames, providing a spatial assessment of phase stability. Figure 5b summarizes the distribution of these standard deviation values as a histogram, offering a comprehensive statistical analysis of the system’s temporal stability over time. Our system achieves an average standard deviation of 85 mrad, demonstrating superior temporal stability compared to traditional off-axis DHM setups, particularly those based on Mach–Zehnder and Michelson interferometer configurations, which are more susceptible to environmental disturbances due to their separate reference and object beam paths. In contrast, our common-path configuration inherently minimizes these fluctuations, making it well-suited for long-term imaging applications, such as biomedical diagnostics, live-cell monitoring, and semiconductor process control.

## Conclusions

We have successfully integrated the meta-biprism into the common-path off-axis DHM system, marking a significant advancement in the field of QPI. The meta-biprism, engineered from GaN nanopillars, serves as a robust and compact beam splitter, enabling a streamlined optical setup that effectively reduces susceptibility to environmental disturbances. Our findings from studies involving resolution targets and human lung cell lines demonstrate the system’s capability to deliver high-contrast, low-noise phase images, allowing for precise thickness measurement down to the nanometer scale. The compact metasurface-based DHM platform is well-suited for point-of-care diagnostics, noninvasive imaging, and portable optical systems. Its compatibility with multimodal imaging approaches, such as fluorescence or polarization imaging, further extends its utility in tissue pathology, drug screening, and personalized medicine.

Conventional biprisms are typically fabricated through grinding and polishing processes, which become increasingly challenging and complex as the element size decreases, particularly in the development of miniaturized optical systems. In contrast, the meta-biprism leverages mature semiconductor fabrication processes, enabling precise nanostructure patterning at extremely small scales. This approach not only facilitates high-volume production and long-term reliability but also ensures the feasibility of integrating metasurfaces into compact optical systems, making them a strong candidate for widespread adoption. Furthermore, the meta-biprism, unlike traditional diffractive gratings that produce undesired higher-order diffraction, leverages engineered subwavelength structures for precise phase control and efficient beam splitting, making it a superior choice for compact, high-performance optical systems.

Additionally, metasurfaces enable asymmetric beam splitting, allowing them to be designed to accommodate light with different angles of incidence, making them adaptable to specific imaging conditions, such as a folded optical system for compact setups. By tailoring the nanostructure arrangement, metasurfaces can also introduce additional phase shifts at the same beam-splitting angle, making them suitable for phase-shifting digital holography. Moreover, metasurfaces can be designed with polarization-selective functionality, enabling applications in multiplexed holography and polarization-selective imaging. This capability allows for the simultaneous measurement of different physical properties of samples, such as birefringence or optical activity, which is particularly advantageous in biological and materials science applications, where polarization analysis can reveal structural and compositional details. Beyond digital holography, the meta-biprism design can be utilized in various optical experiments. For example, it could be employed to generate two identical light sources for quantum optics interference experiments, which traditionally require bulkier and more complex systems.

Looking ahead, future research could focus on enhancing spatial and temporal resolution, integrating multiwavelength capabilities, and incorporating AI-driven image processing for real-time analysis. By leveraging metasurface technology, these developments will push the boundaries of DHM, enabling deeper insights into biological structures and processes.

## Methods

### Numerical Simulations

The design of the unit cell is conducted using CST Microwave Studio, employing the finite integral technique. The structure comprises cylindrical GaN nanopillars, each with a height of 850 nm, arranged on a sapphire substrate with a periodicity of 280 nm. A plane wave with a wavelength of 633 nm, polarized along either the *x*- or *y*-axis, is incident from the substrate side to illuminate the nanopillars. Periodic boundary conditions are applied along the *x* and *y* axes, while an open boundary condition is utilized in the *z* direction. The phase shifts and power transmission are analyzed by varying the diameters of the nanopillars between 120 and 240 nm.

#### Fabrication of Metasurface

The GaN meta-biprism is fabricated using a conventional electron beam lithography technique, followed by ICP-RIE processes. The pattern is generated in a PMMA A4 electron beam resist by using an electron beam writer (Elionix ELS-HS50) on the SiO_2_/GaN/sapphire substrate. This pattern is then transferred to a chromium mask using a lift-off process. The GaN nanopillars are then formed through a two-step RIE process, followed by the removal of the chromium and SiO_2_ masks using chromium etchant and buffered oxide etch (BOE) solution, respectively.

## Supporting Information Available

Beam profile measurement of meta-biprism; detailed schematic of the proposed metasurface-based common-path off-axis DHM system; simulated interference pattern of two beams under different offset angles from 0.2 to 2°; simulated hologram under different offset angles between signal and reference beams, ranging from 0.2 to 2°; simulated spectrum under different offset angles between signal and reference beam, ranging from 0.2 to 2°; phase compensation method; and depth reconstruction by angular spectrum method (PDF)

## Abbreviations

DHM: digital holographic microscopy
ZPC: Zernike phase contrast
DIC: differential interference contrast
QPI: quantitative phase imaging
qDPC: quantitative differential phase contrast
FPM: Fourier ptychographic microscopy
TIE: transport-of-intensity equation
CGM: cross-grating wavefront microscopy
DPM: diffraction phase microscopy
DC: direct current
SEM: scanning electron microscopy
ICP-RIE: inductively coupled plasma reactive ion
AFM: atomic force microscopy
PBS: phosphate-buffered saline
BOE: buffered oxide etch
