# Supplementary material for: Quantitative Phase Imaging with a Meta-Based Interferometric System
Source: ACS Appl Mater Interfaces. 2025 Apr 15;17(17):26023–31. doi: 10.1021/acsami.5c02901 (PMC12051180; doi:10.1021/acsami.5c02901)
Supplement: Supplementary file 1 — am5c02901_si_001.pdf [file am5c02901_si_001.pdf]

Supporting Information

Quantitative phase imaging with a meta-based interferometric system

Cheng Hung Chu[1‡], *Chen-Ming Tsai*[2‡], *Takeshi Yamaguchi*[3], *Yu-Xiang Wang*[4], *Takuo Tanaka*[3,5], *Huei-Wen Chen*[6,7], *Yuan Luo*[1,2,4,8*], *and Din Ping Tsai*[9,10,11*]

[1]YongLin Institute of Health, National Taiwan University, Taipei 10672, Taiwan
[2]Institute of Medical Device and Imaging, National Taiwan University, Taipei 10051, Taiwan
[3]Innovative Photon Manipulation Research Team, RIKEN Center for Advanced Photonics, Saitama 351-0198, Japan
[4]Program for Precision Health and Intelligent Medicine, National Taiwan University, Taipei, 106319, Taiwan
[5]Metamaterials Laboratory, RIKEN Cluster for Pioneering Research, Saitama 351-0198, Japan
[6]Graduate Institute of Toxicology, College of Medicine, National Taiwan University, Taipei 100, Taiwan
[7]Genome and Systems Biology Degree Program, National Taiwan University and Academia Sinica, Taipei 100, Taiwan.
[8]Institute of Biomedical Engineering, National Taiwan University, Taipei 10051, Taiwan
[9]Department of Electrical Engineering, City University of Hong Kong, Kowloon 999077, Hong Kong SAR
[10]Centre for Biosystems, Neuroscience, and Nanotechnology, City University of Hong Kong, Kowloon 999077, Hong Kong SAR
[11]The State Key Laboratory of Terahertz and Millimeter Waves, City University of Hong Kong, Kowloon 99907, Hong Kong SAR.

*Corresponding authors' e-mail addresses: yuanluo@ntu.edu.tw (Yuan Luo), and dptsai@cityu.edu.hk (Din Ping Tsai)

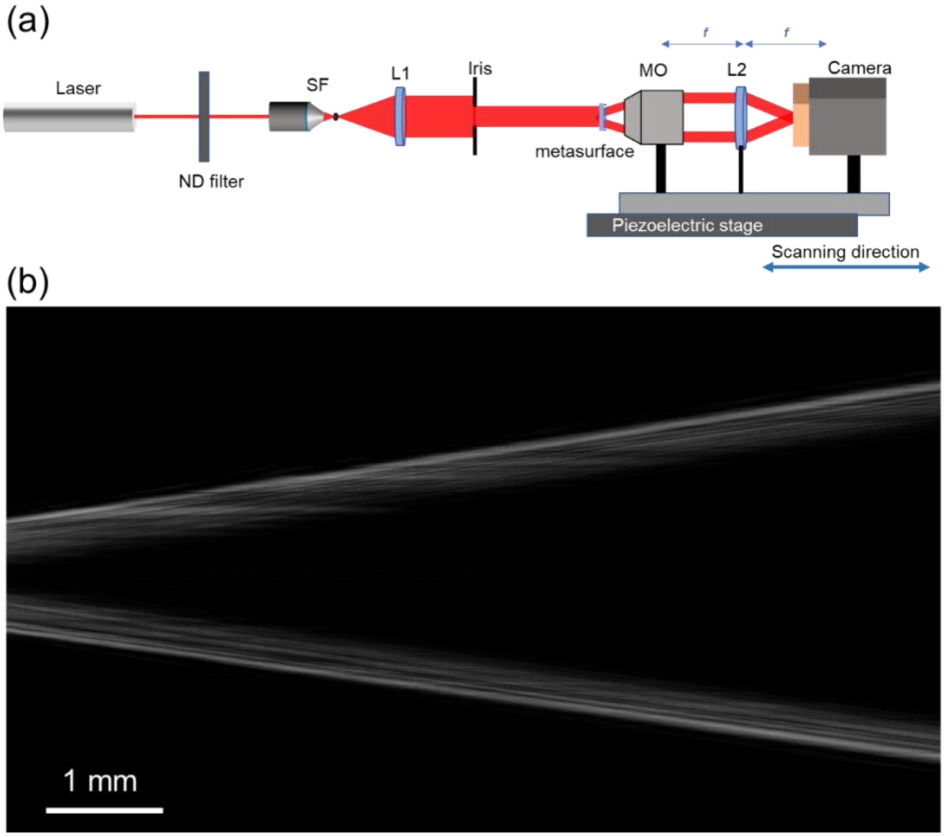

Figure S1. Beam profile measurement of meta-biprism. CCD: charge-coupled device, SF: spatial filter; MO: microscope objective; L: lens.

Fig. S1(a) shows the schematic of the experimental setup used to characterize the beam separation performance of the meta-biprism. A laser beam at the wavelength of 633 nm is passed through a spatial filter and pinhole to enhance beam uniformity. An objective and tube lens are arranged in a 4-f configuration to project onto a camera as an imaging system. The imaging system scans along the beam path to capture the intensity distribution. Fig. S1(b) displays the measured intensity distribution of the meta-biprism in free space.

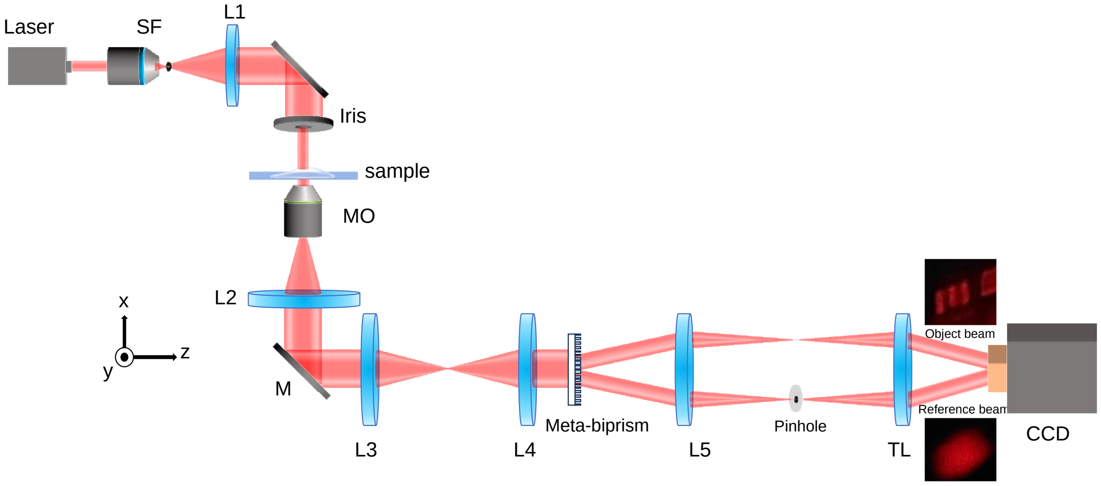

Figure S2. Schematic of the proposed metasurface-based common-path off-axis DHM system. CCD: charge-coupled device, SF: spatial filter; MO, microscope objective; L: lens; TL: tube lens.

A collimated beam at the wavelength of 633 nm, expanded to ensure uniform illumination, is directed through the sample and subsequently collected by a microscope objective. This beam, carrying the information of the scattered complex field of the sample, is then incident on the meta-biprism. The meta-biprism is strategically positioned in the image plane. The meta-biprism separates the incident wavefront into object and reference beams at distinct angles. The reference is filtered at the Fourier plane by using a pinhole to remove any object information from the beam. The two beams then pass through a tube lens and intersect at the CCD sensor, producing an interference pattern.

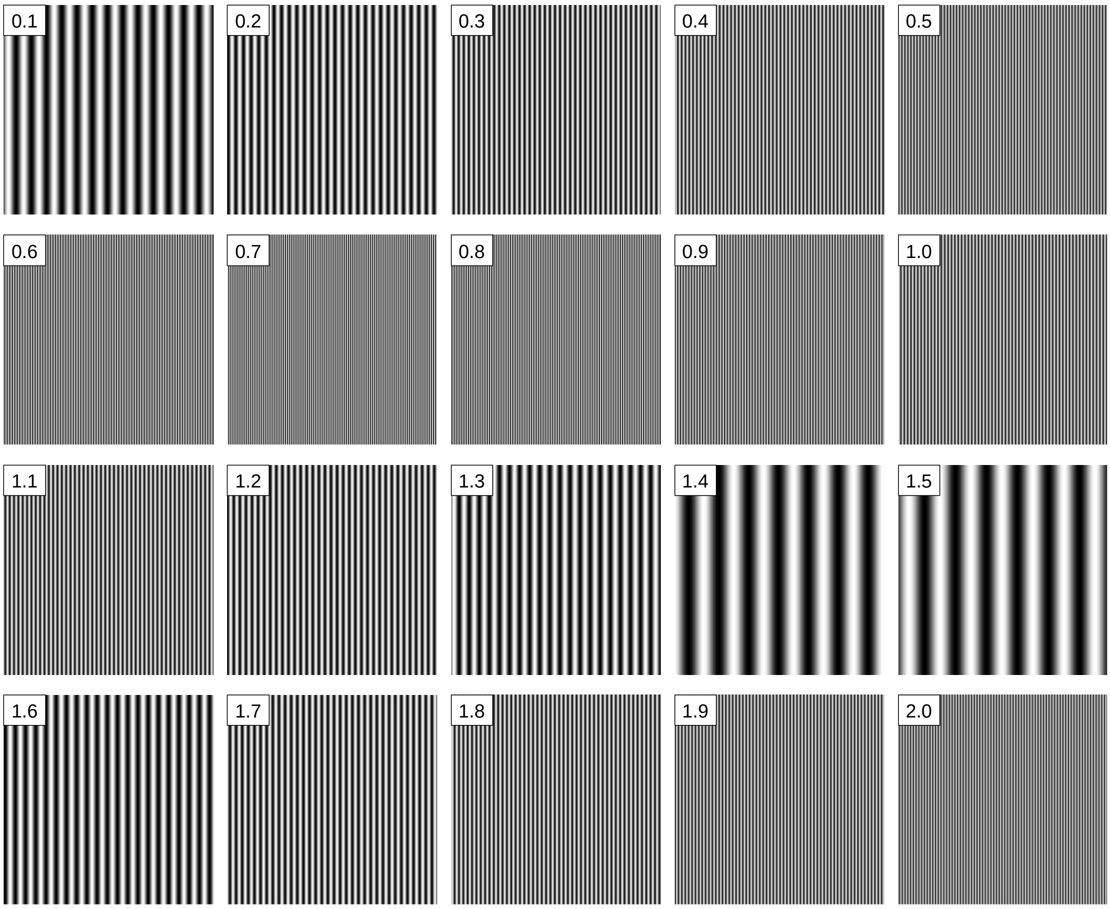

Figure S3. Simulated interference pattern of two beams under different offset angles from 0.2° to 2°.

This figure shows the effect of varying offset angles between two beams ranges from 0.2° to 2° on the frequency of fringe patterns using simulations. These simulations are not intended to match the exact conditions of the experimental setup but rather to demonstrate how variations in offset angles affect spatial frequency distribution and reconstruction quality. At smaller offset angles (e.g., 0.2°), the modulation frequency of the fringes is low, resulting in a sparsely spaced fringe pattern. In the frequency spectrum, the separation between the signal orders is minimal, making it challenging to isolate specific components.

As the offset angle increases, the modulation frequency rises, leading to denser fringe patterns and greater separation between different spectral orders, which improves signal separation. However, the system's ability to record high-density fringes is constrained by the camera's sampling rate, defined by the pixel size of the sensor. When the modulation frequency exceeds the Nyquist frequency, aliasing occurs. This limitation is crucial in DHM system design to prevent aliasing and ensure accurate fringe representation for high-resolution phase imaging.

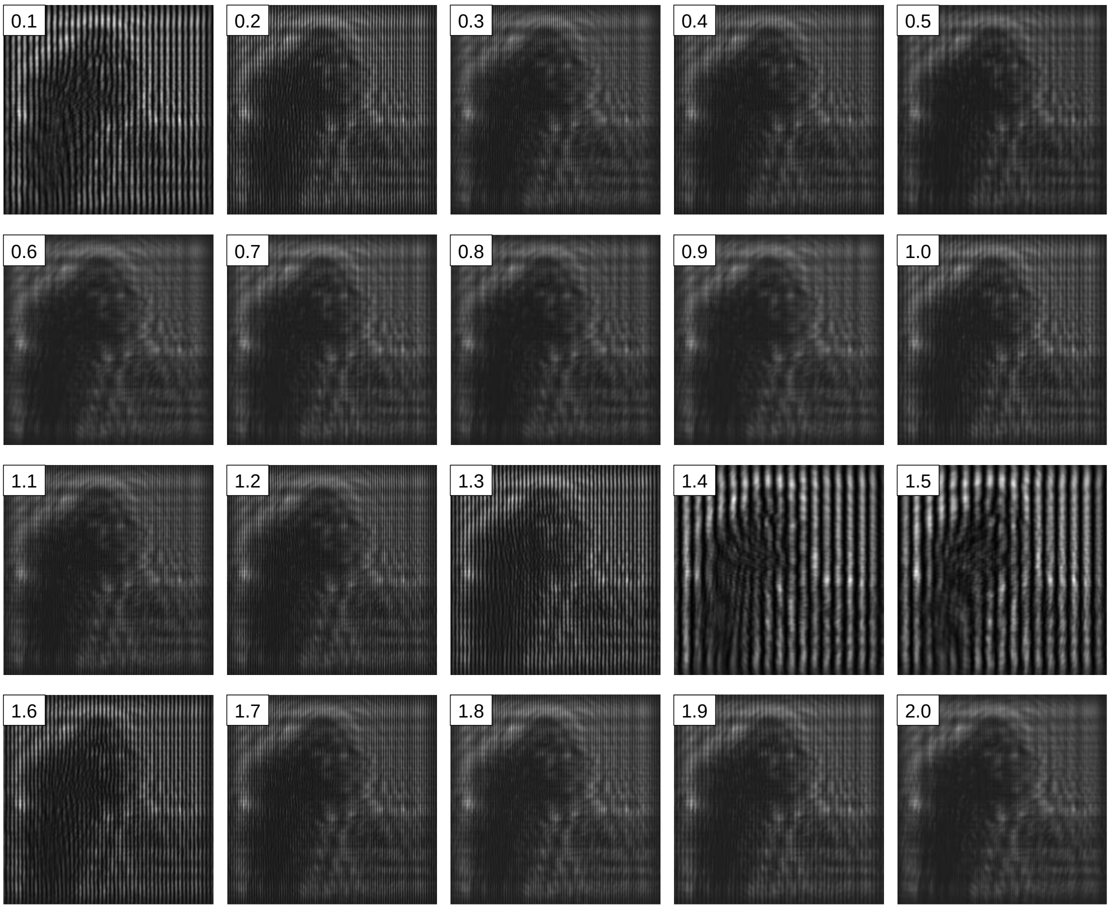

Figure S4. Simulated hologram under different offset angles between signal and reference beams, ranging from 0.2° to 2°.

This figure shows simulated holograms of the "cameraman" image as a test object, chosen for its variety of high- and low-frequency details. As in Figure S3, increasing the offset angle between the signal and reference beams results in higher frequency and denser interference fringes. However, as the fringe density increases, aliasing issues may arise, potentially distorting the hologram at larger offset angles.

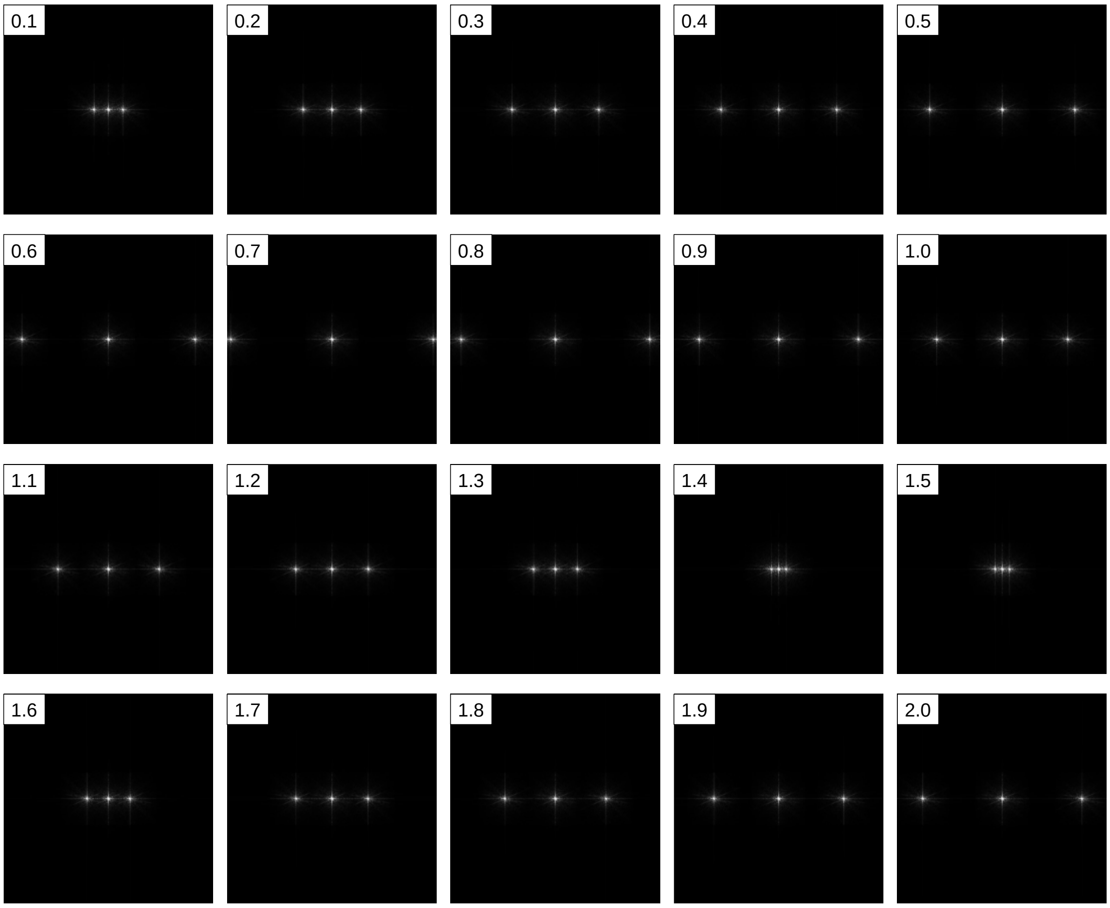

Figure S5. Simulated spectrum under different offset angles between signal and reference beam, ranging from 0.2° to 2°.

This figure shows the simulated spectrum converted from Fig. S4. Different offset angles between the signal beam and reference beam result in varying degrees of signal separation in the frequency domain. The aliasing causes high-frequency components to be misrepresented at lower frequencies, resulting in signal orders appearing closer together in the frequency spectrum.

In the Fourier transform reconstruction process, the region of 'window operation' for filtering the first-order signal is defined by the system's numerical aperture (NA). The

bandwidth of the first-order signal is determined by kNA, while the frequency period is given by $\frac{2\pi}{pNM_{total}}$, where p is the CCD pixel size, N is the total number of pixels in the camera, and M_{total} is the total system magnification [1]. Based on this relationship, the radius of the filter window, in terms of the number of pixels, can be calculated to completely filter the first-order frequency spectrum.

Since kNA represents the finest sample frequency that the system can resolve, setting the initial filter window size to encompass the entire kNA range ensures that the filter remains effective. Consequently, even when measuring objects with different phase distributions, there is no need to adjust the size of the filter window.

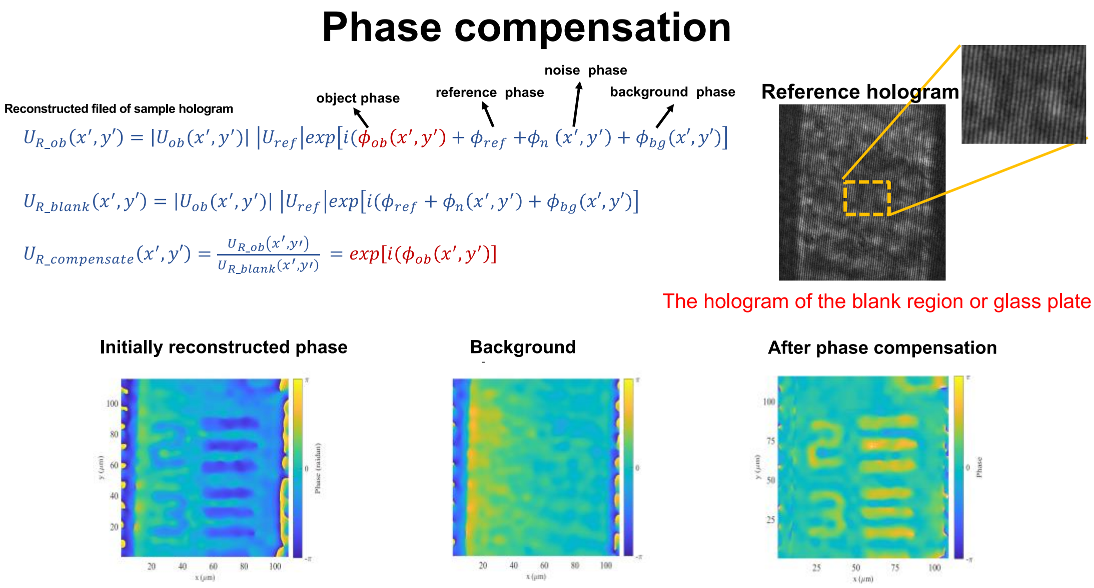

Figure S6. Phase compensation method.

In digital holographic microscopy (DHM), accurately reconstructing the phase map of the hologram requires compensating for background and noise effects, which may distort phase measurements. This figure illustrates the mathematical approach used for phase compensation, along with visual examples of the method applied to experimental data. $U_{R\_ob}(x', y')$ represents the reconstructed field of the object, containing both the object's phase information and unwanted phase contributions from the background and noise sources. The background phase $U_{R\_blank}(x', y')$ is separately measured by capturing a hologram of a blank region or glass plate without the object in place. The phase-compensated image $U_{R\_compensate}(x', y')$ is computed by dividing the object field by the background field, effectively isolating the sample's intrinsic phase $\phi_{ob}(x', y')$ and removing background contributions.

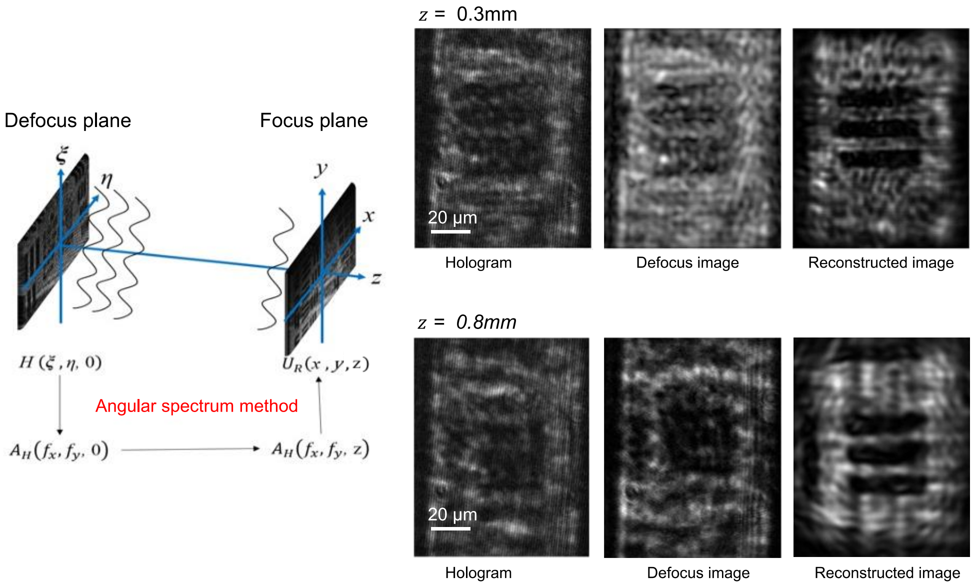

Figure S7. Depth reconstruction by angular spectrum method. The left panel illustrates the principle of depth reconstruction by utilizing the angular spectrum method (ASM). The right panel displays reconstructed images at two distinct defocused depths. The top row shows hologram and defocus images at z = 0.3 mm and the corresponding reconstructed focus image at z = 0 mm. The bottom row shows hologram and defocus images at z = 0.8 mm and the corresponding reconstructed focus image at z = 0 mm.

An initial hologram $H(\xi, \eta, 0)$ is captured at the defocus plane, encoding both amplitude and phase information of the wavefront emanating from the sample. The hologram is transformed into the spatial frequency domain by applying a two-dimensional Fourier transform, yielding its angular spectrum $A_H(f_x, f_y, 0)$. This spectrum represents the distribution of plane waves with different propagation angles in the hologram. The

wavefront at the defocus plane is propagated to the focal plane by introducing a phase shift in the frequency domain, which accounts for the distance z between planes. Mathematically, the phase shift is represented as spatial frequency transfer function, $\exp(i2\pi z\sqrt{\frac{1}{\lambda^2} - {f_x}^2 - {f_y}^2})$, where λ is the wavelength of the light used. This step effectively simulates the propagation of the wavefront through free space, allowing the reconstruction at the desired focal depth. Finally, an inverse Fourier transform is applied to bring the propagated angular spectrum $A_H(f_x, f_y, z)$ back to the spatial domain, producing the reconstructed image $U_R(x,y,z)$ at the focal plane.

Reference:

[1] B. Bhaduri, C. Edwards, H. Pham, et al., "Diffraction phase microscopy: principles and applications in materials and life sciences," Advances in Optics and Photonics **6**, 57-119 (2014).
